# Data Prediction of Mobile Network Traffic in Public Scenes by SOS-*v*SVR Method

**DOI:** 10.3390/s20030603

**Published:** 2020-01-22

**Authors:** Xiaoliang Zheng, Wenhao Lai, Hualiang Chen, Shen Fang

**Affiliations:** 1 State Key Laboratory of Mining Response and Disaster Prevention and Control in Deep Coal Mines, Anhui University of Science and Technology, Huainan 232000, China; zhengxl@aust.edu.cn; 2 School of Electrical and Information Engineering, Anhui University of Science and Technology, Huainan 232000, China; hualiang_chen520@163.com; 3 Huainan Branch of China Mobile Group Anhui Company Limited, Huainan 232000, China; fangshen@ah.chinamobile.com

**Keywords:** public scene, mobile network traffic data prediction, *v*SVR, symbiotic organisms search

## Abstract

Accurate base station traffic data in a public place with large changes in the amount of people could help predict the occurrence of network congestion, which would allow us to effectively allocate network resources. This is of great significance for festival network support, routine maintenance, and resource scheduling. However, there are a few related reports on base station traffic prediction, especially base station traffic prediction in public scenes with fluctuations in people flow. This study proposes a public scene traffic data prediction method, which is based on a  v Support Vector Regression (*v*SVR) algorithm. To achieve optimal prediction of traffic, a symbiotic organisms search (SOS) was adopted to optimize the *v*SVR parameters. Meanwhile, the optimal input time step was determined through a large number of experiments. Experimental data was obtained at the base station of Huainan Wanda Plaza, in the Anhui province of China, for three months, with the granularity being one hour. To verify the predictive performance of *v*SVR, the classic regression algorithm extreme learning machine (ELM) and variational Bayesian Linear Regression (vBLR) were used. Their optimal prediction results were compared with *v*SVR predictions. Experimental results show that the prediction results from SOS-*v*SVR were the best. Outcomes of this study could provide guidance for preventing network congestion and improving the user experience.

## 1. Introduction

Mobile Internet not only enriches people’s entertainment life, but also brings great convenience to daily life. The relationship between our life and mobile network has become closer. By the end of March 2019, the number of 4G users in China was 1.204 billion. The per capita traffic was 7.27 GB per month [1]. Also, the use number and per capita traffic maintain a growing trend. Rapidly growing network size and user demands present many challenges to current network infrastructure. For some multifarious personnel flow and densely populated scenes (high-speed rail stations, tourist attractions, commercial centers, playgrounds, sports competitions, concert venues, etc.), the rapidly growing traffic puts tremendous pressure on its network architecture. Burst traffic data of a wireless network may cause the failure of the normal function of the base station equipment. When that problem is not severe, users’ internet access speed just becomes slow; however, in extreme cases, users cannot access the internet and the mobile phone connection rate drops sharply. People often try to connect to network under such failure circumstances, which will bring secondary shocks to wireless networks and core networks, e.g., at around 11:08 on 29 May 2019, there was a brief interruption in 4G internet access of batch users in municipalities of China. Then some users re-registered, causing instantaneously high load work to seven sets of switches. Some mobile phones under switches could not access the internet. The number of users affected by this network accident exceeded one million.

In daily life, traffic-change prediction based on hourly granularity and guidance for on-demand allocation of network resources could reduce network operation costs. In festivals or large-scale activities, the user size and mobile traffic of public scenes could be predicted quickly and accurately based on people flow and the tidal effect of traffic. This prediction could help operators master upcoming congestion and make network expansion, adjustment, and optimization in advance, and use limited wireless carrier resources to meet network peaks. Emergency plan for a network schedule could developed to give access to users that does not degrade in case of sudden increased traffic. Under the impact of large-scale network congestion and blocking, traffic and routing could be scheduled in time to ensure the network access rate and network connection rate of essential areas and to retain connected customers. Therefore, the prediction of traffic data in a base station for multifarious personnel flow and densely populated public scenes is of considerable significance in network security.

At present, there are related reports on base station traffic prediction [2,3,4,5,6]. However, research on traffic prediction for 4G base stations in public scenes has seldom been reported. For example, the authors of [2] extracted and modeled traffic patterns of 9000 cellular towers deployed in a metropolis, and evaluated a big data-driven method for predicting mobile network traffic data. However, this method was only for traffic prediction of large-scale cellular networks. In [3], an end-to-end deep Traffic Predictor (TP) based on deep learning was proposed. This realized traffic demand prediction based on data in Shanghai and predicted base station traffic data from third-generation communication technology (3G). A deep TP requires massive training data, has higher requirements for hardware. Besides, and deep models are more difficult to train. Qiu et al. [4] proposed a wavelet-based stacked denoising autoencoder (Wavelet-SDA) deep learning framework to predict the number of users of mobile base stations; however, it was not used to predict traffic data. Therefore, this paper proposes a traffic data prediction model for 4G networks based on machine learning in public places, which is used for traffic prediction of public scenes with large changes in people flow. This study aims to provide guidance for operators’ network security and maintenance.

Traffic prediction is a typical time series prediction problem, also known as a regression problem in machine learning [7]. Commonly used regression algorithms are the autoregressive integrated moving average model (ARIMA) [8], Bayesian linear regression (BLR) [9], extreme learning machine (ELM) [10], support vector regression (SVR) [11], artificial neural network (ANN) [12,13,14,15], et al. SVR was developed by Vapnik’s theory of the support vector machine [16]. From a theoretical point of view, the principle of structural risk minimization (SRM) in SVM makes it have better generalization power than the principle of empirical risk minimization (ERM) used in neural networks [17]. SVR has great potential and excellent performance in practical applications. For example, the wind speed prediction of SVR wind farms is reported in [18]. Results showed that comparing with the least-squares method for autoregressive model adjustment, SVR has a higher accuracy for wind energy technology (WET) prediction. Kaytez et al. [19] comprehensively compared neural networks with the least squares support vector machine in predicting electricity consumption in Turkey. Analysis results showed that the Least Squares-Support Vector Machines (LS-SVM) model can be effectively used for long-term net power consumption predictions in Turkey. Chen et al. [20] used seasonal index adjustment SVR for vacation forecasting of daily tourist traffic. Results showed that SVR had a higher accuracy and less errors than the Back Propagation Neural Network (BPNN) model. In addition, SVR was successfully applied to a toxicity assessment [21], battery life prediction [22,23], chemical production [24,25], financial projection [26,27], and agricultural production [28,29]. Based on excellent regression performance and a wide range of successful application cases of SVR, it was selected to predict the traffic data of the 4G network base stations in public scenes in this study.

Symbiotic organisms search (SOS), a new metaheuristic optimization algorithm proposed by Cheng et al. [30], which was inspired by symbiotic relationship between organisms in the ecosystem. A significant advantage of SOS is that, it does not have any specific adjusting parameters, avoiding risk of incorrect parameter adjustments which may affect result accuracy. The algorithm implementation is simple and stable. SOS optimization has been tested on selected benchmark functions. Results were compared with many recently developed effective optimization techniques, such as genetic algorithm (GA), bees algorithm (BA), particle swarm optimization (PSO), and differential evolution (DE). Results showed that SOS had a better performance than other metaheuristic technologies [31]. As a result, the SOS algorithm has received extensive attention in the optimization research community and other related fields [32]. Ezugwu et al. [33] used SOS for the optimization of travelling salesmen’s problems and the discrete search performance of SOS was further verified. Cheng et al. [34] allocated multiple resources in the process of SOS optimization of multiple project-planning problems (DSOS-MRLMP). Results indicated that the SOS model could obtain more reliable and effective results. Verma et al. [35] used SOS for congestion management in a deregulated environment and Çelik et al. [36] used SOS for the design of the Proportional Integral Derivative (PID) controller.

Although SVR has excellent optimization performance, it is as sensitive to parameter settings as other machine learning algorithms. Manual determination of optimal parameters of SVR is time-consuming and laborious. Based on the excellent optimization performance of SOS, we used the symbiotic organisms search optimization algorithm for parameter optimization of SVR, which was used for traffic data prediction of 4G network base stations in public scenes. In this study, we not only used ELM and vocational Bayesian Linear Regression (vBLR) for traffic prediction, but also adopted moth-flame optimization (MFO) [37] and PSO for optimization. Finally, the results were used for optimization algorithm comparison and performance verification of SOS-SVR. 

## 2. Materials and Methods

### 2.1. Methods

#### 2.1.1. v Support Vector Regression Algorithm

Support vector regression is an application of a support vector machine in a regression task. Support vector machine (SVM) [16,38] is a classical algorithm that is driven from results of statistical learning theory. It was originally developed for classification tasks in the field of pattern recognition and represented decision boundaries of a typically small subset of all training examples called support vectors. The core task of SVM is to find the hyperplane with the largest interval, then divide the samples into different categories. 

SVM and SVR function is described as: f(x)=w·Φ(x)+b, where b is the bias and w is weight vector. Φ is nonlinear mapping, *x* is mapped into a feature space in which a linear estimate function is defined. $x\in R^{N}$, $R^{N}$ is represents input space.

Given a data $\left(x_{i},y_{i}\right)$, $x_{i}$ and $y_{i}\in R$, the constrained form is:

(1) $$\begin{array}{l} \min\frac{1}{2}{\left\|w\right\|}^{2}\\ s.t.\;y_{i}\cdot (W^{T}x_{i}+b)\geq 1,\;\forall i \end{array}$$

where, *W* is an element in the high-dimensional spaces, *b* is the threshold, ${\left\|w\right\|}^{2}$ is the description function.

The core of SVR is to find a hyperplane to minimize structural risks. To reduce the effect of noise, a slack variable ξ is introduced. Thus, the constraint becomes:

(2) $$\begin{array}{l} \min\frac{1}{2}{\left\|w\right\|}^{2}+C\sum_{i=1}^{m}\left(\xi_{i}+{\hat{\xi }}_{i}\right)\\ \;\begin{array}{c} s.t.\; \end{array}\;\left\{\begin{array}{l} y_{i}-\left(W^{T}x_{i}+b\right)\leq \varepsilon +\xi_{i}\\ \left(W^{T}x_{i}+b\right)-y_{i}\geq \varepsilon +{\hat{\xi }}_{i} \end{array}\right.\;,\;\xi_{i},{\hat{\xi }}_{i}\geq 0,w\in W \end{array}$$

where, C is a constant, ε is insensitive loss function.

Equation (2) is a constrained optimization problem. When a Lagrange function is introduced, and a dual form is obtained, the maximization function is as follows:

(3) $$\begin{array}{l} W\left(\alpha ,\hat{\alpha }\right)=\sum_{i}^{m}\left(\alpha_{i}-{\hat{\alpha }}_{i}\right)y_{i}-\varepsilon \sum_{i}^{m}\left(\alpha_{i}+{\hat{\alpha }}_{i}\right)-\frac{1}{2}\sum_{i}^{m}\left(\alpha_{i}-{\hat{\alpha }}_{i}\right)\left(\alpha_{j}-{\hat{\alpha }}_{j}\right)\left(x_{i}\cdot x_{j}\right)\\ \begin{array}{c} s.t. \end{array}\;\left\{\begin{array}{l} \sum_{i}^{m}\left(\alpha_{i}-{\hat{\alpha }}_{i}\right)=0\\ 0\leq \alpha_{i},\;\alpha_{i}^{*}\leq C \end{array}\right. \end{array}$$

where, α is a Lagrangian operator.

The regression estimate obtained by Equation (3) is linear. In order to adapt the support vector regression to a nonlinear problem, an input vector is mapped into a high-dimensional feature space (in Equation (4)), then is re-performed in this space. Linear regression is used to obtain effect of nonlinear regression in original space.

(4) $$x_{i}\cdot x_{j}\to \Phi \left(x_{i}\right)\cdot \Phi \left(x_{j}\right)$$

where, $\Phi \left(\cdot \right)$ is a mapping function that maps *x* from low-dimensional space to high-dimensional space, which is also called a kernel function. 

When solving ε Support Vector Regression (εSVR), the parameter ε plays a decisive role in fitting performance of objective function. However, it is difficult to determine the reasonable value. In order to determine the reasonable value of ε more conveniently, a new parameter v denoted as *v*SVR is introduced to control number of support vectors and training error. Therefore, its constraints change accordingly:

(5) $$\begin{array}{l} \min\frac{1}{2}{\left\|w\right\|}^{2}+C\left(v\varepsilon +\sum_{i=1}^{m}\left(\xi_{i}+{\hat{\xi }}_{i}\right)\right)\\ s.t.\;\left\{\begin{array}{l} y_{i}-\left(W^{T}x_{i}+b\right)\leq \varepsilon +\xi_{i}\\ \left(W^{T}x_{i}+b\right)-y_{i}\geq \varepsilon +{\hat{\xi }}_{i} \end{array}\right.\;,\;\xi_{i},{\hat{\xi }}_{i}\geq 0 \end{array}$$

where, the value range of *v* is [0, 1]. The dual problem of Lagrange function of *v*SVR constraint is shown in Equation (6):

(6) $$\begin{array}{l} W\left(\alpha ,\hat{\alpha }\right)=\sum_{i}^{m}\left(\alpha_{i}-{\hat{\alpha }}_{i}\right)y_{i}-\frac{1}{2}\sum_{i}^{m}\left(\alpha_{i}-{\hat{\alpha }}_{i}\right)\left(\alpha_{j}-{\hat{\alpha }}_{j}\right)k\left(x_{i},x_{j}\right)\\ \begin{array}{c} s.t. \end{array}\;\left\{\begin{array}{l} \sum_{i}^{m}\left(\alpha_{i}-{\hat{\alpha }}_{i}\right)=0,\;\sum_{i}^{m}\left(\alpha_{i}+{\hat{\alpha }}_{i}\right)\leq Cv\\ 0\leq \alpha_{i},\;\alpha_{i}^{*}\leq C \end{array}\right. \end{array}$$

where, $k\left(x_{i},x_{j}\right)$ is the mapping of input vectors to high-dimensional space. The kernel function is a Gaussian kernel function. By solving above optimization problem, we could finally get the output function of support vector machine:

(7) $$f\left(x\right)=\sum_{i}^{m}\left(\alpha_{i}-{\hat{\alpha }}_{i}\right)\cdot k\left(x_{i},x_{j}\right)+b$$

##### 2.1.2. Symbiotic Organisms Search

Symbiotic organisms search [30] is a new meta-heuristic swarm intelligence optimization algorithm proposed by Cheng et al. and was inspired from the symbiotic interactions observed between two organisms in the ecosystem.

(1). Symbiosis

In 1869, German mycologist de Bary first used this word, symbiosis, to define the relationship between two different species of organisms that are interdependent. Symbiotic relationships are broadly divided into two types, such as obligate and facultative. In an obligate relationship, both organisms entirely depend on each other for their survival, whereas in a facultative relationship, the organisms may depend on each other, but it is not mandatory. Three types of symbiotic relationships are found in nature. These are mutualism, commensalism, and parasitism. Mutualism refers to the relationship between two different species of organisms where both individuals are benefited. Commensalism describes the symbiotic relationship between two organisms in which one benefits and the other is, not significantly, affected. Parasitism is the kind of symbiotic relationship where one organism is benefited and the other is, effectively, harmed. Living organisms undergo symbiotic relationships in order to adapt themselves in the environment and, hence, they improve their fitness to survive in the ecosystem over the long term.

(2). Features

SOS algorithms commence with an initial population of organisms which is called the ecosystem. Almost all metaheuristic algorithms apply a succession of operations to solutions in each iteration in order to generate new solutions for the next iteration. In SOS, new solution generation is governed by imitating the biological interaction between two organisms in the ecosystem. Each organism of the ecosystem is considered as a candidate solution to the corresponding problem and is correlated to a certain fitness value which imitates the degree of adaptation to the desired objective. The new solutions are generated by simulating the symbiotic interactions between two organisms in the ecosystem, which includes the mutualism, commensalism and parasitism phases. Each organism in the ecosystem randomly interacts with the other through all these three phases and this process of interaction is repeated until the termination criterion is fulfilled.

(3). Mutualism phase

In this phase, the relationship established by the mutually beneficial symbiotic order can benefit both sides of the organism. A similar example is a relationship between bees and flowers; the organism renewal formula is as shown in Equation (8):

(8) $$\left\{\begin{array}{l} x_{inew}=x_{i}+r_{1}\cdot (x_{best}-\varphi \cdot BF_{1})\\ x_{jnew}=x_{j}+r_{2}\cdot (x_{best}-\varphi \cdot BF_{2})\\ \varphi =0.5\left(x_{i}+x_{j}\right) \end{array}\right.$$

where, $r_{1}$ and $r_{2}$ are rand number in [0, 1], $x_{i}$ and $x_{j}$ are two organisms that have a symbiotic relationship, φ is the relationship characteristics between the two organisms. BF represents the level of benefit of the two organisms, recorded as the benefit factor. In some symbiotic relationships, the benefits obtained by the two organisms are different.

1. Commensalism Phase

In this phase, the relationship established during the commensal phase benefits only one organism, while the other is harmless or has little interest. A similar example is the relationship between crucians and a shark. The organism at this stage is shown:

(9) $$x_{inew}=x_{i}+r_{3}\cdot (x_{best}-x_{j})$$

where, $r_{3}$ is a random number in [0, 1], $x_{i}$ is the party that benefits more from the symbiotic relationship and $x_{j}$ is the party that is harmless in this relationship.

2. Parasitism Phase

In this phase, one organism gets benefits and the other organism is damaged. A similar example is the relationship between malarial mosquitoes and humans. In the SOS algorithm, this stage forms an individual called "Parasite_Vector" by duplicating the organism $x_{i}$, then modifying the randomly selected dimensions using a random number. The Organism $x_{j}$ is selected randomly from the ecosystem and serves as a host to parasite vector. Parasite_Vector tries to replace $x_{j}$ in the ecosystem. Both organisms are then evaluated to measure their fitness. If Parasite_Vector has a better fitness value, it will kill the organism $x_{j}$ and assume its position in the ecosystem. If the fitness value of $x_{j}$ is better, $x_{j}$ will have immunity from the parasite. Parasite_Vector will no longer be able to live in that ecosystem. The optimization process of SOS is as follows:

Step 1: Define and initialize the ecosystem population, and initialize all variables or parameters;
Step 2: Find the best individual $x_{best}$ in the population;
Step 3: $x_{i}$ and $x_{j}$ are selected randomly. Calculate the modified organism according to Equation (8), calculate the fitness value, and update the individual information;
Step 4: $x_{j}$ is selected randomly. Calculate the modified organism according to Equation (9), calculate the fitness value, and update the individual information;
Step 5: $x_{i}$ and $x_{j}$ are selected randomly. Modify the values of certain dimensions of organism *i*, creating a Parasite_Vector. Calculate the fitness value, compare it with the organism *j*, and choose to update or retain;
Step 6: If the current creature is not the last creature in the ecosystem, go back to step 3;
Step 7: Identify the end condition of the search. If no, go back to step 2; If yes, output the optimal results.

##### 2.1.3. SOS-vSVR Algorithm 

If the parameters can be set reasonably, *v*-SVR will have excellent performance in regression tasks. These parameters include the regularization coefficient *C* in Equation (6) and number parameter *v* for controlling support vector. In addition, the kernel function used in this study is Gaussian kernel function (in Equation (10)), and the parameters g also need reasonable settings.

(10) $$k\left(x_{i},x_{j}\right)=\exp(-g{\left\|x_{i},x_{j}\right\|}^{2})$$

**Figure 1:**
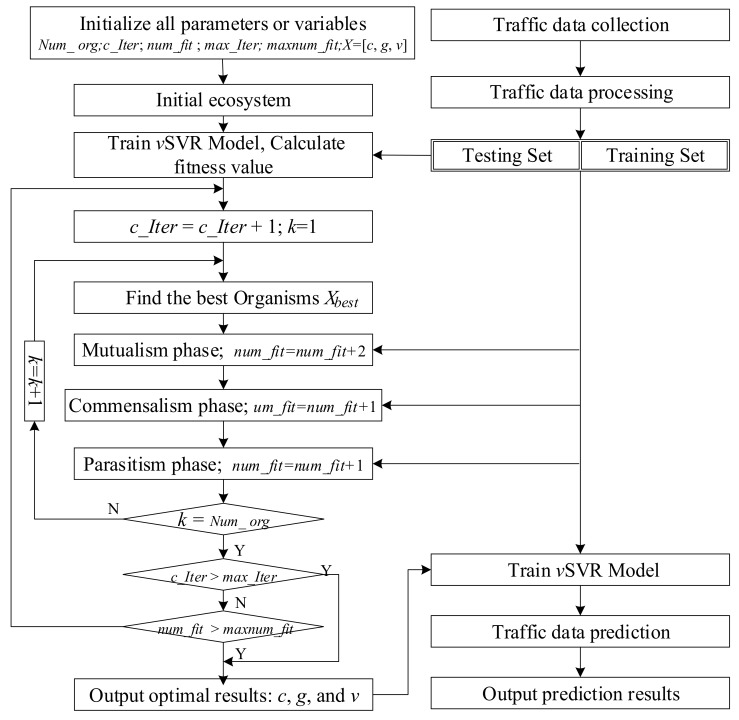
The flowchart of symbiotic organisms search (SOS) optimized μ support vector regression (*v*SVR).

Manually setting of parameters *c*, *g*, and *v* requires lots of experimentation, which is time-consuming and difficult to find the optimal value. Therefore, SOS is used for the optimization of *v*SVR parameters as follows:

In Figure 1, *Num_org*, *c_Iter*, *num_fit*, *max_Iter*, and *maxnum_fit* are the number of creatures, the current number of iterations, the number of fitness calculations in optimization process, the maximum number of iterations, and the maximum number of calculations in optimization process, respectively. Root mean square error (RMSE) was used to calculate the fitness value, as shown in Equation (11):

(11) $$RMSE\;=\;\sqrt{\;\frac{1}{n}\sum_{i=1}^{n}{\left|y_{i}-\hat{y}\right|}^{2}\;}$$

where, $y_{i}$ and ${\hat{y}}_{i}$ are observations and predictions, respectively.

#### 2.2. Materials

##### 2.2.1. Experimental Data Collection

**Figure 2:**
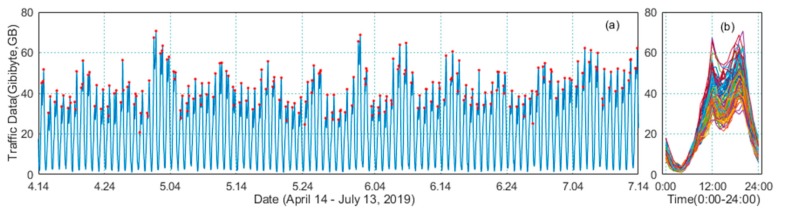
Base station data of Wanda Plaza. (**a**) Data curve for April 14–July 13. (**b**) Daily traffic comparison.

In this paper, the selected public scene was Huainan Wanda Plaza. The period for obtaining traffic data was 14 April–13 July 2019, including International Labor Day (May 1) and China’s traditional festival Dragon Boat Festival (June 7). The obtained traffic data is shown in Figure 2.

In Figure 2, the data collection granularity is 1 h, that is, 24 times per day. It could be seen from the figure that the daily traffic flow trend is similar (the peak value is usually the data collected at 12:00 or 20:00). However, the daily peak values vary greatly, and the festivals’ traffic peaks were significantly higher than the non-holiday traffic peaks. Among the obtained traffic data, the maximum daily traffic peak was on May 1 (holiday), which was 70.67 GB, while the minimum daily peak value was on May 22 (Wednesday, weekday), which was 35.11 GB.

##### 2.2.2. Data Processing

Base station data prediction is a typical time series prediction problem. Data processing for time series prediction usually has two parts, one is numerical processing (such as data normalization), and the other is sequence processing (processed into equal length input data and its corresponding output data).

(12) $$x^{\prime }=\frac{x}{\max\left(X\right)}\;,\;x\in X$$

where $x^{\prime }$ is the processed traffic data.

If we use a normalized method to process the data, we cannot directly use the prediction results to evaluate the model because there is a zero value in the normalized data $y_{i}$. In this paper, we used Equation (12) to process traffic data in numerical values. Compared to the normalized processing method, a significant advantage of Equation (12) is that it allows us to directly use prediction results to evaluate the prediction performance of the model without inverse transformation.

**Figure 3:**
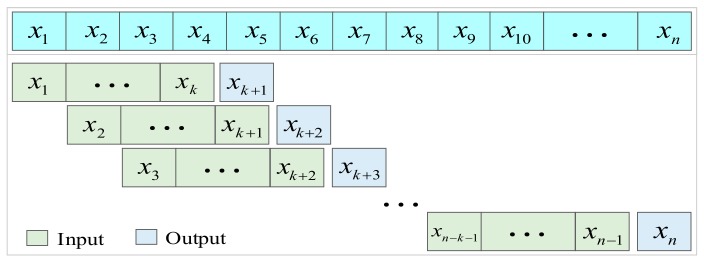
Sequence processing of traffic data.

For sequence processing, the data is sliced into sequences of equal length, and each sequence is continuous. Equal-length sequences are used as inputs to the prediction model. The next value of each sequence is output as the training model target. The way of sequence processing of traffic data is as shown in Figure 3.

In Figure 3, *n* and *k* are the length of time series and the length of flow data of theinput prediction model, respectively. The model output length is 1. After sequence processing, the input data has *n*-*k* samples, that is, the input data dimension is (*n*−*k*) *k. In this paper, the data collected were from 14 April to 13 July 2019.24 data were collected each day, so the value of *n* is 2184.

The length of input data affects the *v*SVR different performance (*k* takes different values). In this paper, to get the best prediction, the optimization of *k* was one of our main tasks.

## 3. The Prediction of Traffic Data

### 3.1. Experimental Preparation

#### 3.1.1. Experiments Setup

In machine learning, when the data is not extremely large, the rate of training set to the test set is generally 4:1. Therefore, the rate of training set and test set in this paper is 4:1. The traffic data from May 2 to July 13 was used for the training of the predicted model, while the remaining data was used for the model test. To verify the performance of SOS optimized *v*SVR, we also used the optimization algorithms MFO and PSO for parameters optimization of *v*SVR. Besides, to test the performance of *v*SVR on traffic data prediction, εSVR, ELM, and vBLR were also used for prediction. The above prediction results are used for comparison. In the experiment, the parameter settings of each optimization algorithm are shown in Table 1.

In Table 1, the variables *ub_cgv_* and *lb_cgv_* are the upper and lower boundary vectors of the parameters *c*, *g*, and *v* of the *v*SVR algorithm, respectively. The variables *ub_cg_* and *lb_cg_* are the boundary vectors of parameters *c* and *g* of the εSVR algorithm, respectively.

Each experiment in this paper was run many times, independently. All experiments in this work were conducted on a personal computer with Intel(R) Core (TM) i7-9700K processor, 3.60 GHz, 16GB RAM, using Windows 10 as the operating system. The LibSVM-3.23 implementation was used for the SVR regression. We also used Matlab implementation environment in our experiment.

#### 3.1.2. Prediction Model Evaluation Method

In the parameter optimization process of prediction algorithm, the fitness function of optimization algorithm used RMSE. To more objectively compare the performance of different prediction algorithms, mean absolute percent error (MAPE) was also used to evaluate the model. The evaluation mechanism of MAPE is shown in Equation (13).

(13) $$MAPE\;=\;\frac{1}{n}\sum_{i=1}^{n}\left|\frac{y_{i}-{\hat{y}}_{i}}{y_{i}}\right|\;\times 100\%$$

where, $y_{i}$ and ${\hat{y}}_{i}$ are observations and predictions, respectively.

### 3.2. Application of SOS-vSVR in Traffic Data Prediction 

In this part of experiment, we used SOS to search for the optimal parameters of *v*SVR, and then used the obtained optimal parameters for traffic data prediction. To verify the optimization performance of SOS and the performance of *v*SVR, we not only used MFO and PSO for parameter optimization of *v*SVR, but also used εSVR for traffic data prediction.

**Figure 4:**
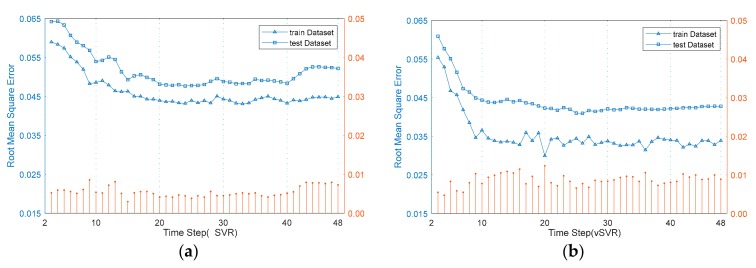
Root mean square error (RMSE) of *v*SVR and εSVR when *k* gets different values. (**a**) The RMSE of *v*SVR; (**b**) The RMSE of εSVR.

In the time series prediction problem, the sequence length of model input affects the prediction performance. The optimal sequence length corresponding to different prediction models may also be different. To better obtain the best performance of SVR on traffic data prediction in a public scene, we first needed to get the optimal sequence length, that is, the optimal *k* value. For different values of *k*, the minimum RMSE of *v*SVR and εSVR searched by SOS are shown in Figure 4.

In the experiment, each *k* value was repeated ten times, independently. The minimum RMSE was selected. In Figure 3, the left axis is RMSE of training set and test set, while the right axis is the subtraction of RMSE of test set and training set.

In an excellent classification or regression model, the error in training set and test set both should be small. Likewise, in an excellent classification or regression model, the test error is as small as a training error, but it is usually higher than the training error. Therefore, we were more concerned with the performance of the prediction model on the test set. It can be seen from Figure 4 that when the *k* values are the same, the RMSE of the *v*SVR is less than the RMSE of εSVR, whether it is the training error or test error. Besides, the minimum RMSE of the vSVR and εSVR on the test set decreased first, before increasing with the rise of *k*. The minimum RMSE of *v*SVR and εSVR on the test set are 0.0409 and 0.0476, respectively, and the corresponding *k* values are 26 and 24, respectively. Therefore, in the next part of experiment, the length of *v*SVR and εSVR input sequences were 26 and 24. The results of SOS, MFO, and PSO optimization of vSVR and εSVR are shown in Table 2.

In Table 2, each model was repeatedly optimized ten times, and the optimal results were selected. c, g, and v are the parameter values corresponding to optimal predictions of *v*SVR and εSVR searched by the optimization algorithm. ‘Optimization time’ is the average time of 10 repeated experiments.

It can be seen from Table 2 that the support vector predicted base station traffic data in the public scene. The prediction accuracy of *v*SVR was significantly better than εSVR, whether on the training set or the test set. SOS, MFO, and PSO were used to optimize *v*SVR parameters, to predict base station traffic data for public scenarios. The prediction results of SOS-*v*SVR on the training set were optimal, whether it was RMSE or MAPE, and the prediction result of MFO-*v*SVR on the test set was optimal.

In the experiment, different optimization algorithms were used to search the parameters of *v*SVR, the searched optimal settings were used to predict the base station traffic data of the public scene. The optimal prediction results differed in different data sets and evaluation methods. However, compared to the difference between the predicted results of εSVR and *v*SVR, the difference between the optimal results of the optimization algorithm search was very small. For example, the maximum difference in the MAPE of the *v*SVR searched by SOS, MFO, and PSO on the test set was 0.18%, and the maximum difference in MAPE between εSVR and *v*SVR was 8.32%.

SOS, MFO, and PSO optimize *v*SVR to predict traffic data. In terms of accuracy, MFO and SOS had the same excellent performance. However, SOS had an absolute advantage in optimizing time-consumption, whether it was optimizing εSVR or *v*SVR. For example, the average time of SOS optimization εSVR was less than one-tenth of MFO, which means that SOS optimization efficiency is very high in traffic prediction.

### 3.3. Compared With Non-Optimal Regression Algorithms

In the previous section, we used PSO and MFO for support vector regression optimization and compared the results with SOS optimization results to verify the efficiency of SOS-*v*SVR in traffic prediction. In this section, extreme learning machine (ELM) and variational Bayesian linear regression (vBLR) are adopted to predict mobile traffic, which in turn validates the advancement of *v*SVR’s predicted traffic data.

We optimized the *v*SVR when it predicted traffic data. For fairness, the best results of ELM and vBLR for traffic prediction should also be obtained before comparison. In this part of experiment, we first determined the optimal time step *k* of ELM and vBLR. Since the regression performance of ELM is closely related to the number of hidden layer nodes. It is also necessary to determine the optimal number of hidden layer nodes of ELM.

**Figure 5:**
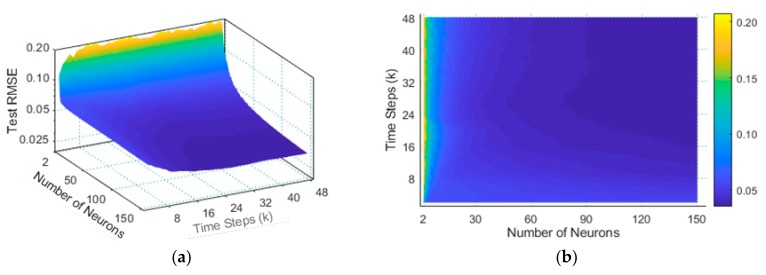
RMSE of *v*SVR and εSVR when *k* takes different values. (**a**) The RMSE of *v*SVR; (**b**) The RMSE of εSVR.

In Figure 5, the value of *k* is an integer in [2, 48], and the number of hidden layer nodes is an integer of [2, 150]. Since some of the ELM parameters are random, and these random parameters also affect their performance. When we looked for the optimal parameters of ELM, our number of experiments was as high as 47*149*100 times (the combination of different *k* and hidden layer neuron numbers was repeated 100 times independently).

As can be seen from Figure 5b, when the time step was greater than 20 and the number of hidden layer nodes was greater than 90, the error of ELM on the test set no longer decreased with the increase in number of nodes. Considering computer resources and efficiency, we set the number of hidden layer nodes of the ELM to 90. Also, it can be seen from Figure 5, the ELM predicted the traffic data of public scene base station. The influence of the number of hidden layer nodes on prediction performance was greater than the time step *k*.

**Figure 6:**
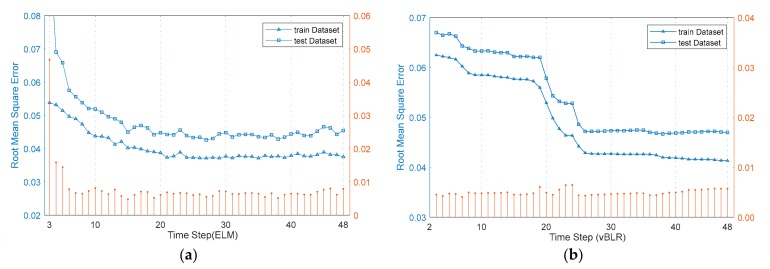
RMSE of ELM and vBLR when k takes different values. (**a**) The RMSE of ELM; (**b**) The RMSE of *v*SVR.

Figure 6a shows the training and test error curves for different time steps when the number of hidden nodes in the ELM was 90, and Figure 6b is the error curve for vBLR. As can be seen from Figure 6, the value of *k* corresponding to the minimum RMSE of ELM on the test set is 27, while the value of *k* corresponding to the minimum RMSE of vBLR is 27. The minimum RMSE of the ELM and vBLR on the test set are 0.0426 and 0.0472, respectively.

**Figure 7:**
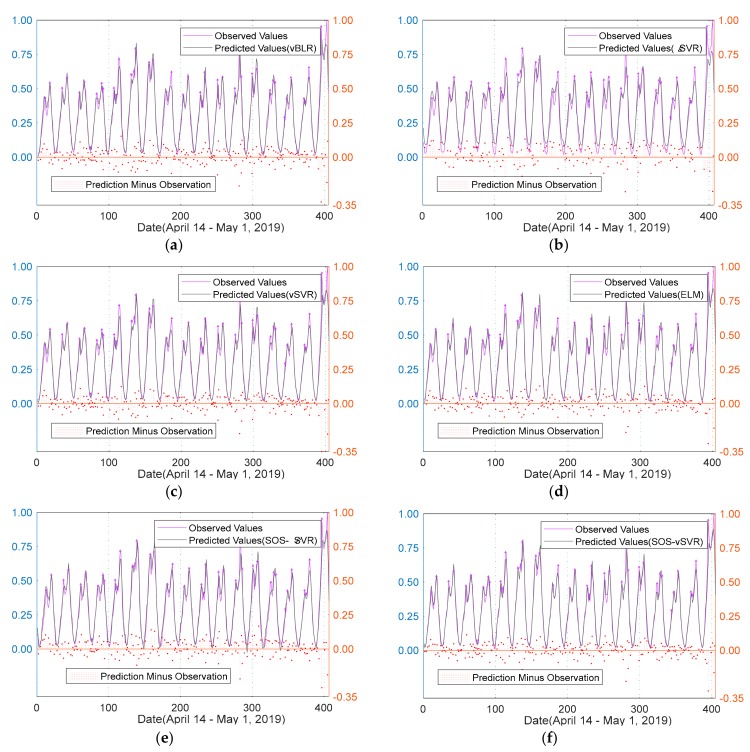
Comparison of predicted and observed values of each regression model on the test set. (**a**) vBLR. (**b**) εSVR (**c**) *v*SVR. (**d**) ELM. (**e**)SOS-εSVR. (**f**) SOS-*v*SVR.

The RMSE and MAPE comparisons of ELM, vBLR, and SOS-SVR are shown in Table 3. Figure 7 is a comparison of predicted and actual values for each regression model on the test set.

In Table 3, “εSVR” and “SOS-εSVR” represent the settings of default parameters of εSVR and search optimal values, respectively. The “*v*SVR” parameter setting is a default value. The minimum MAPE of εSVR and *v*SVR with default parameters on the test set was 43.41% and 14.23%, qfter parameter optimization, the MAPE was 20.61% and 12.36%, respectively. This means that after the parameter optimization, the prediction accuracy of support vector machine for traffic data was improved. Especially the εSVR regression, the error rate of traffic prediction was reduced by more than 20% after SOS parameter optimization. ELM and vBLR predicted traffic data. The MAPE on the test set was 14.35% and 15.98%, respectively, which were higher than the MAPE of SOS-*v*SVR. Compared with the ELM and vBLR regression algorithms, whether it is RMSE or MAPE, the prediction results of SOS-*v*SVR for public scene base station data were optimal.

In Figure 7, the left coordinate is the comparison between prediction result of the regression algorithm and actual value. While the right coordinate axis is the subtraction of the predicted value and observed value. From Figure 6, we can more intuitively see that the SOS-*v*SVR prediction results were optimal.

## 4. Conclusions

Traffic prediction of the base station in a public scene is of great significance for network emergency support during festivals and large-scale activities. This paper takes Huainan Wanda Plaza as the experimental object and established a *v*SVR traffic prediction model. To achieve optimal traffic prediction, we not only used SOS for *v*SVR parameter optimization, but also optimized the time step. In the experiment, when the time step was 26, the RMSE and MAPE of *v*SVR optimal traffic prediction on test set were 0.0409 and 12.36%, respectively, and the corresponding parameters c, g, and v were 3.28, 0.2602, and 0.837, respectively.

To verify the superiority of SOS-*v*SVR in traffic prediction, first, we also used MFO, PSO, and εSVR in experiments for comparison. Results showed that SOS optimizes *v*SVR parameters with the highest efficiency. Second, ELM and vBLR were adopted to predict traffic data. For fairness, a large number of experiments were used to obtain optimal predictions of traffic data from ELM and vBLR. Results showed that SOS-*v*SVR has the least error in traffic prediction, whether it is a test set or a training set.

## Figures and Tables

**Table 1:** Initial parameters of the SOS, particle swarm optimization (PSO), and moth-flame optimization (MFO).

Algorithm	Parameter	Value
SOS	*max_Iter*;	30
	*maxnum_fit*	100
	Number of organisms	40
PSO	Acceleration constants	[1.7; 1.5]
	Inertia w	[0.9, 0.75]
	Generations	30
	Number of particles	40
MFO	*b*	1
	Iterations	30
	Number of search agents	40
SOS/PSO /MFO-*v*SVR	*lb_cgv_*	[0.1; 0.001; 0.01]
	*ub_cgv_*	[100; 10; 1]
SOS/PSO /MFO-εSVR	*lb_cg_*	[0.1; 0.001]
	*ub_cg_*	[100; 10]

**Table 2:** Prediction results of *v*SVR and εSV.

Algorithm	Time Steps (*k*)	Train		Test		*c*	*g*	*v*	Optimization Time (s)
		RMSE	MAPE	RMSE	MAPE				
SOS-*v*SVR	26	0.0332	9.25%	0.0409	12.36%	3.28	0.2602	0.837	298.25
MFO-*v*SVR	26	0.0333	9.43%	0.0408	12.19%	5.84	0.1969	0.776	1433.11
PSO-*v*SVR	26	0.0346	9.81%	0.0415	12.37%	57.12	0.0557	0.891	1189.03
SOS-εSVR	24	0.0432	17.38%	0.0476	20.61%	63.73	0.0692	—	2.94
MFO-εSVR	24	0.0432	17.43%	0.0475	20.54%	100.00	0.0568	—	30.37
PSO-εSVR	24	0.0432	17.30%	0.0476	20.51%	64.83	0.0675	—	23.16

Abbreviation: MAPE, mean absolute percent error.

**Table 3:** Prediction results of SVR, vBLR, and ELM.

Algorithm	Optimal Time Step (*k*)	Train		Test	
		RMSE	MAPE	RMSE	MAPE
vBLR	26	0.0429	13.79%	0.0472	15.98%
ELM	27	0.0371	12.42%	0.0426	14.35%
εSVR	24	0.0554	39.05%	0.0624	43.41%
SOS-εSVR	24	0.0432	17.38%	0.0476	20.61%
*v*SVR	26	0.0402	12.25%	0.0457	14.23%
SOS-*v*SVR	26	0.0332	9.25%	0.0409	12.36%
